# Emerging approaches to CDK inhibitor development, a structural perspective

**DOI:** 10.1039/d2cb00201a

**Published:** 2022-12-14

**Authors:** Ian Hope, Jane A. Endicott, Jessica E. Watt

**Affiliations:** a Translational and Clinical Research Institute, Faculty of Medical Sciences, Newcastle University, Paul O’Gorman Building, Framlington Place Newcastle upon Tyne NE2 4HH UK Jessica.Watt@newcastle.ac.uk

## Abstract

Aberrant activity of the cyclin-dependent kinase family is frequently noted in a number of diseases identifying them as potential targets for drug development. However, current CDK inhibitors lack specificity owing to the high sequence and structural conservation of the ATP binding cleft across family members, highlighting the necessity of finding novel modes of CDK inhibition. The wealth of structural information regarding CDK assemblies and inhibitor complexes derived from X-ray crystallographic studies has been recently complemented through the use of cryo-electron microscopy. These recent advances have provided insights into the functional roles and regulatory mechanisms of CDKs and their interaction partners. This review explores the conformational malleability of the CDK subunit, the importance of SLiM recognition sites in CDK complexes, the progress made in chemically induced CDK degradation and how these studies can contribute to CDK inhibitor design. Additionally, fragment-based drug discovery can be utilised to identify small molecules that bind to allosteric sites on the CDK surface employing interactions which mimic those of native protein–protein interactions. These recent structural advances in CDK inhibitor mechanisms and in chemical probes which do not occupy the orthosteric ATP binding site can provide important insights for targeted CDK therapies.

## Abstract 2

This review summarises recent developments in structural characterisation of CDKs and alternative non-ATP competitive ways to inhibit them.

## Graphical abstract

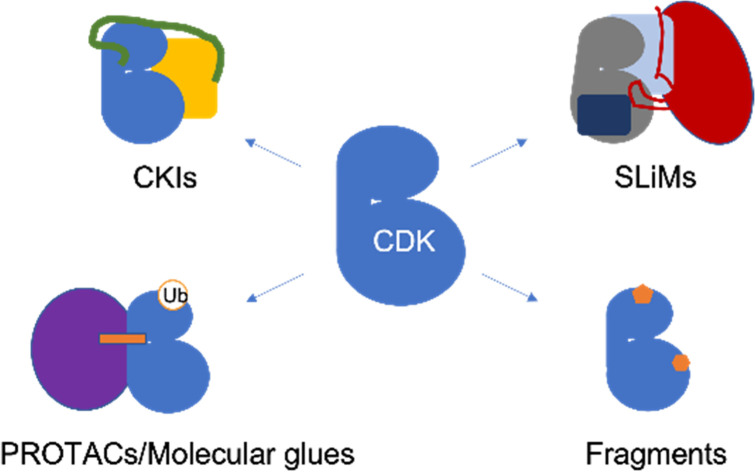

## 1. Introduction

Cyclin-dependent kinase (CDK) targeted drug discovery strategies have predominantly used biochemical, activity-based assays coupled with structural insight to improve inhibitor potency and selectivity. This approach has aided the design and development of many ATP-competitive small molecules (Type I and II)^1,2^ though these inhibitors can exhibit issues with selectivity and off-target effects due to the high degree of conservation of the ATP pocket within the CDK family. Recently, CDK inhibitor studies have shifted focus to explore the structure–function relationship of CDK-containing multimeric complexes. These complexes are regulated by post-translational modifications, protein–protein and protein–ligand interactions. Such events result in specific conformations that modulate CDK activity. Characterisation of similarities and differences between monomeric CDKs and larger CDK-containing assemblies can improve understanding of the protein–protein interactions (PPIs) and the mechanistic allostery involved in CDK regulation.

Dysregulation of the cell cycle or transcription to promote tumorigenesis is frequently a consequence of over- or reduced expression or mutation of CDKs, cyclins, cyclin-dependent kinase inhibitors (CKIs) or other components of CDK-containing complexes. Mutations in proteins that sit more distant from CDKs in regulatory pathways may reveal unanticipated molecular dependencies that are cell lineage specific. These biological and disease relevant CDK sub-populations may have favourable druggable pockets and/or targetable dynamics and therefore offer more opportunity to identify allosteric and PPI inhibitors with improved selectivity and alternative mechanisms of action. Collectively, they provide a compelling mechanistic rationale to continue to target CDKs.^3–8^

Twenty proteins are now categorised as members of the CDK family, a large proportion of which have been extensively characterised as key regulators of the cell cycle, transcription, metabolism and/or cell differentiation.^9,10^ Studies of other CDKs, such as CDK10, 11 14–18 and 20 have evidenced more specific roles and continue to enrich the CDK literature.^11–14^ CDK expression levels remain relatively stable throughout the cell cycle and their activation is primarily driven by association with a cyclin partner. For CDKs that regulate cell cycle progression, their cognate cyclin partners display a characteristic oscillating expression pattern which helps to define each stage of the cell cycle. In contrast, the expression levels of cyclins that activate transcriptional CDKs remain high in proliferating cells to support ongoing gene expression.

CDKs 1, 2, 4 and 6 have well characterized functions that collectively regulate the eukaryotic cell cycle. During G1 phase, formation of CDK4/6-cyclin D complexes in response to mitogenic signals phosphorylate and inactivate the retinoblastoma protein (pRb), as well as other G1 proteins.^15–17^ Transcription factor E2F is released from pRb and an increase in expression of E2F-regulated substrates, *e.g.*, cyclin E1, is triggered. CDK2 complexes with cyclin E1 to further phosphorylate and inactivate pRb and stimulate expression of cell cycle-related proteins that promote progression from G1 to S-phase. Cyclin A bound to CDK2 is required for S-phase and then CDK1 bound to cyclin A and subsequently cyclin B drives the cell through G2 and mitosis.^18^ These later cell cycle events are driven by a second wave of gene transcription regulated by CDK activity that disrupts the DREAM repressor to promote the formation of the MYB-MuvB-FOXM1 transcription complex.^19^

The initiation of mRNA synthesis is also an orderly process, characterised by stage-specific transcription factor recruitment and post-translational modifications. The transcriptional CDKs ensure a timely, forward progression of RNA polymerase II (RNAPII) along the gene through phosphorylation of the RNAPII carboxy terminal domain (CTD) and associated transcription factors. CDK7 and CDK8 regulate transcription initiation as constituents of CDK-activating kinase (CAK) and the CDK8-kinase module (CKM) of the mediator complex, respectively.^20–22^ Transcription begins with CDK7 driven phosphorylation of the RNAPII CTD heptapeptide repeats (TyrSerProThrSerProSer) weakening the interaction between RNAPII and core mediator complex (cMED) stimulating a switch from initiation to productive elongation.^23,24^ CAK is tethered to TFIIH *via* the MAT1-XPD interaction which ensures correct positioning of CAK for CTD phosphorylation and transcriptional promoter escape.^25^

CAK is also required for the phosphorylation and activation of other kinases including cell cycle CDKs 1, 2, 4 and 6 and transcriptional kinase CDK9.^26,27^ CAK activates the CDK9 containing positive transcription elongation factor (P-TEFb) to overcome proximal promoter pausing and facilitate transcription elongation by further phosphorylating the RNAPII CTD, the negative elongation factor (NELF) and DRB sensitivity inducting factor (DSIF).^27–31^ RNAPII CTD phosphorylation is sustained by CDK12 and CDK13 during elongation and termination.^32^ CDK11 has also been reported to phosphorylate the CTD and plays a role in pre-mRNA splicing.^13^ The roles of transcriptional CDKs are less understood in detail owing to their presence in these larger macromolecular assemblies and complex PPI networks. However, though challenging to develop, there is mounting evidence to support the therapeutic value of transcriptional inhibitors particularly in cancer.^33–35^

This review discusses recent CDK structural studies (in particular those published since 2018) that have advanced our understanding of CDK-protein interactions.^9^ We explore the contributions of structural flexibility, allostery and short linear motifs (SLiMs) to the regulation of CDK activity and the use of small molecules and chemically-induced targeted CDK degradation to perturb CDK signalling.

## 2. Recent progress in CDK structure–function studies

**Fig. 1:**
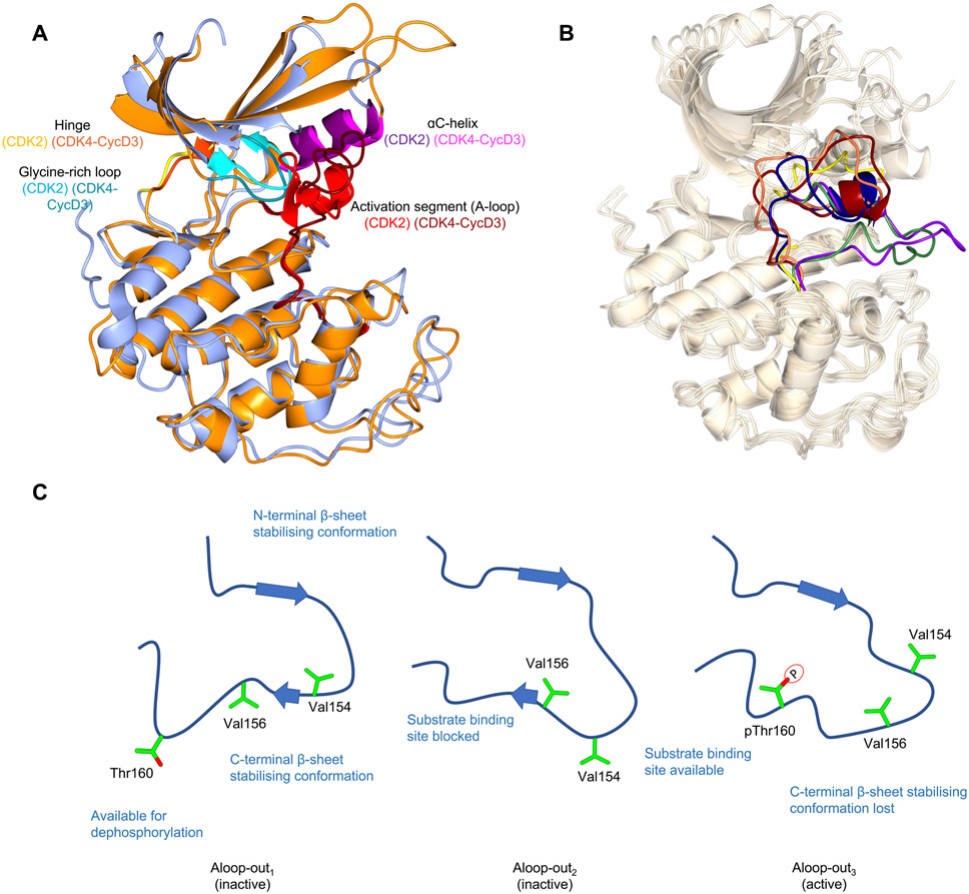
The inactive CDK fold and A-loop intermediates. (A) Overlay of monomeric CDK2 (blue, PDB 1HCK^39^) and CDK4 from a CDK4-cyclin D3 complex (gold, PDB 3G33^40^) as examples of a monomeric and cyclin-bound CDK with an inactive fold. In the inactive conformation, the αC-helix within the N-lobe is positioned outward precluding the formation of a key salt bridge. The glycine lid is oriented downwards towards the active site. A difference between CDK2 and CDK4 is the presence of the αL12 helix in the A-loop of CDK2 which is melted in CDK4, highlighting two of many different conformations the A-loop can adopt. (B) Alternative A-loop conformations as shown by 5 overlaid CDK2 crystal structures (PDBs: CDK2-Cyclin A (1FIN^38^ green), CDK2-2AN (3PXF^56^ dark red), CDK2 (3PXR^56^ dark blue), CDK2-X64 (4GCJ^57^ coral), CDK2-LQ5 (5A14^58^ yellow) and CDK2-Spy1 (5uq2^41^ purple). (C) Three proposed A-loop-out conformations.^62^ Aloop-out_1_ and Aloop-out_2_, typically observed for monomeric CDK2, maintain states incompatible with substrate binding and use β-sheet mediated interactions with the C-terminal lobe (C-lobe) to stabilise the A-loop while facilitating Thr160 access to phosphatases. This C-lobe interaction is lost in Aloop-out_3_ with residues sitting even closer to the cyclin interface with pThr160 interacting with cyclin Arg50, Arg126 and Arg150 (not shown).

Crystal structures of monomeric CDK2 and CDK2-cyclin A complexes provided the first structural evidence for our current models of how mechanistically relevant CDK conformations are enabled by cyclin binding, substrate interactions and phosphorylation.^18,36–38^ Monomeric CDK2 adopts an inactive structure in which the region after the DFG motif of the activation segment (A-loop) is either melted or forms a short single turn helix (αL12) (Fig. 1A).^9,39,40^ This secondary structure coupled with the DFG-out A-loop-in orientation blocks the αC-helix from moving towards the active site, creating an auto-inhibited conformation. When in the active cyclin-bound state, movement inwards of the αC-helix forms a Glu51-Lys33 salt bridge, and the A-loop positions itself outwards adopting a conformation that recognises the peptide substrate subsequent to phosphorylation on T160. Phosphorylation of the A-loop is not required to activate CDK2 when it is bound to RINGO/Speedy family members – meiosis-specific proteins that only encode a single cyclin-box fold (CBF).^41,42^ Here, the A-loop is pulled out of the active site to form a platform that is structurally distinct from that seen in Thr160-phosphorylated CDK2 (pCDK2)-cyclin A but is compatible with peptide substrate recognition and catalysis (Fig. 1B).

αC-helix malleability has been exploited to design type II ATP-competitive inhibitors that target the inactive kinase conformation and remodel the back of the ATP binding pocket. As most CDKs have a bulky phenylalanine as the gatekeeper residue, few Type II CDK inhibitors have been reported, the exception being a series targeting CDK8 for which this sequence changes to DMG (residues 173–175).^43^ ATP-competitive CDK inhibitors have been extensively reviewed.^44–49^

The observation that monomeric CDK1 and CDK2 can be readily distinguished by their ATP-competitive inhibitor binding properties demonstrates that though they are highly conserved in sequence there are significant differences in their conformational energy landscapes that can impact function.^50^ Cyclin-free CDK1 affinities for inhibitors such as dinaciclib,^51^ AZD5438,^52^ flavopiridol,^53^ SU9516^54^ and CGP74514A^55^ are significantly weaker compared to cyclin-free CDK2, unlike their cyclin-bound states which are comparable in affinities. While structurally similar interactions were observed, the difference between monomeric CDK1 and CDK2 may be explained by structural flexibility in the contacts between the N-terminal and C-terminal lobes (N-lobe and C-lobe).^50^ An overlay of monomeric CDK2 crystal structures also reveals the structural diversity in the vicinity of the active site^38,41,56–58^ particularly the alternative conformations that the A-loop can adopt (Fig. 1B). This characteristic is hypothesized to be a conserved feature of monomeric CDK structures, and has been verified for cyclin-free CDK1 (PDB: 4YC3)^59^ and monomeric CDK6 (PDB: 5L2I)^60^ and CDK7 (PDB: 1UA2).^61^

Additional intermediate transition states of monomeric CDK2 have also been characterised using double electron–electron resonance (DEER) and nuclear magnetic resonance spectroscopy experiments (Fig. 1C).^62^ Conformational sampling between A-loop-in and 3 alternative A-loop-out states highlight multiple steps between inactive and active conformations that regulate CDK2 activation. A-loop-out_1_ lowers conformational energy barriers and retains allosteric coupling between the phosphorylation of Thr160 and cyclin binding. This conformation also provides additional regulation by reducing residual kinase activity by adopting a state optimal for phosphatase activity, supported by structures showing both A-loop-in and A-loop-out conformations.^41,56–58^ A comparison between pCDK2, CDK2-cyclin A and pCDK2-cyclin A showed that only pCDK2-cyclin A significantly populated the A-loop-out_3_ conformation in which Thr160 interacts with Arg50, Arg126 and Arg150, as observed in crystal structures of the active complex (PDB: 1W98).^63^ Further work will determine whether the ranges of alternative states monomeric CDKs can adopt offers opportunities to identify novel hotspots for probe development and a structural window to achieve significant selectivity.

CDK4 is unusual in that unlike other structurally-characterised CDKs it remains in an inactive state when cyclin-bound (Fig. 1A).^40,47^ Despite A-loop phosphorylation in the CDK4-cyclin D1 structure, the αC-helix remains out, and the beginning of the A-loop forms an α-helix reminiscent of the αL12 helix found in monomeric CDK2. From these observations it has been proposed that substrate engagement may be an element of the structural mechanism of CDK4-cyclin D activation.^40^

**Fig. 2:**
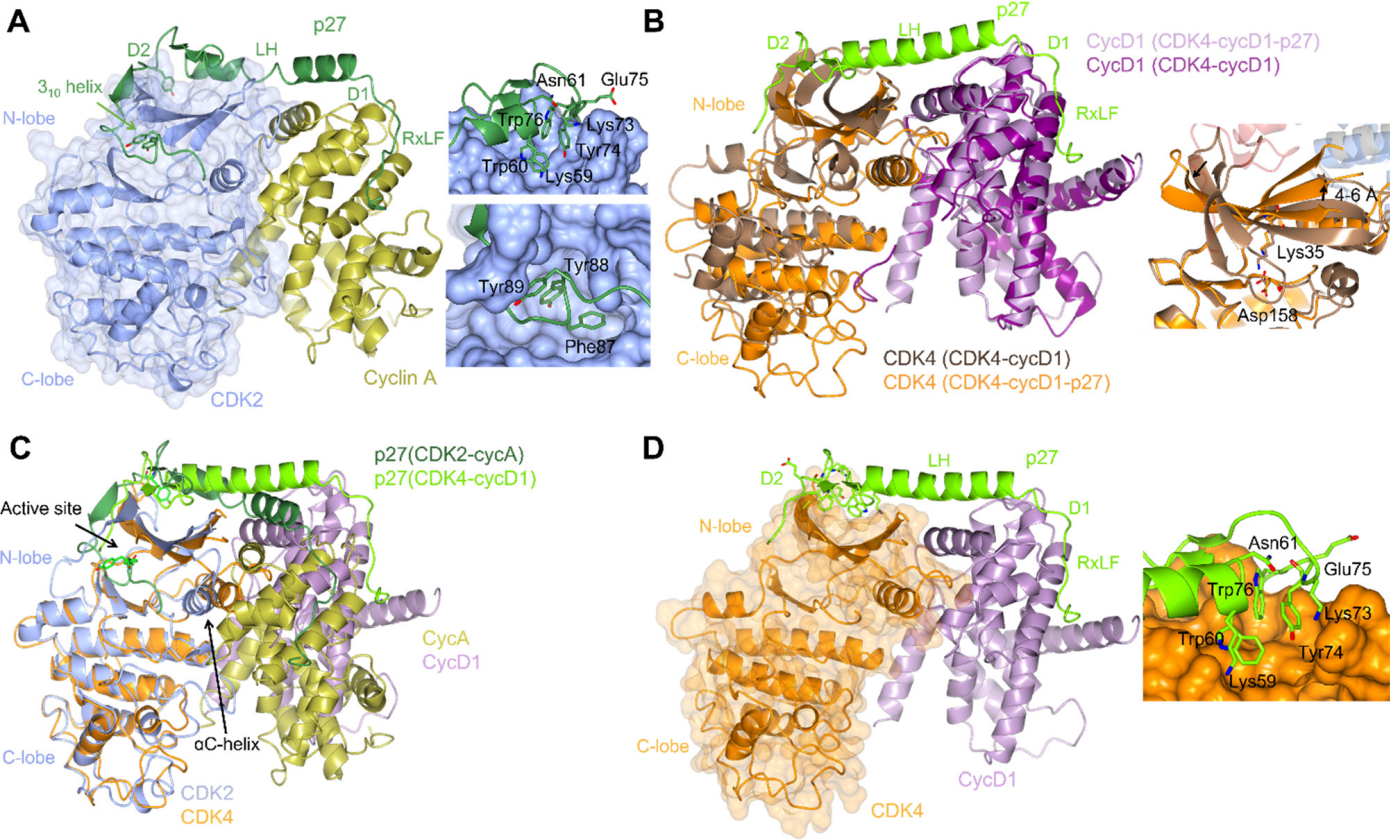
Structural comparison of CDK2-cyclin A-p27 with CDK4-cyclin D1-p27. (A) structure of CDK2-cyclin A-p27 (PDB: 1JSU;^67^ CDK2-blue, cyclin A-gold, p27-dark green) showing p27 wrapping around the RXL site on cyclin A then extending across to interact with the N-lobe of CDK2. Right top panel shows engagement of Tyr74 and neighbouring residues of p27 with the CDK2 N-lobe; right bottom panel shows the occupancy of the CDK2 active site by the p27 3_10_ helix region containing Tyr88. (B) N-lobe rotation in CDK4 that accompanies p27 binding highlighted by an overlay of CDK4-cyclin D1 (PDB: 2W96;^70^ CDK4-brown, cyclin D1-purple) and CDK4-cyclin D1-p27 (PDB: 6P8E;^69^ CDK4-orange, cyclin D1-lilac, p27-lime green). Right panel highlights the change in the position of Lys35 and the interaction with Asp158. (C) Overlay of CDK2-cyclin A-p27 (PDB: 1JSU;^67^ CDK2-blue, cyclin A-gold, p27-dark green) with CDK4-cyclin D1-p27 (PDB: 6P8E;^69^ CDK4-orange, cyclin D1-lilac, p27-lime green) showing the differences in the positions of the αC-helix and the CDK N-lobes. (D) Structure of CDK4-cyclin D1-p27 (PDB: 6P8E;^69^ CDK4-orange, cyclin D1-lilac, p27-lime green) showing the residues interacting between the D2 region of p27 and N-lobe of CDK4.

An alternative mechanism to promote CDK4 cyclin assembly and activation is through association with members of the CIP/KIP family of CDK inhibitors, p21^CIP1^ and p27^KIP1^ (hereafter p21 and p27 respectively).^64–66^ This role is in apparent contradiction to their well-established function as CDK inhibitors, first structurally characterised following the determination of a CDK2-cyclin A-p27 complex (Fig. 2A).^38,67^

p21 and p27 are classed as intrinsically disordered proteins and fold upon binding to CDK-cyclin complexes.^37,64,65^ To act as an inhibitor, exposure of the p27 D1 region (residues 27–37) which contains an RXL motif that binds to the cyclin recruitment site (Fig. 2A) primes binding of the p27 kinase inhibitory domain (KID, residues 28–89) *via* conformational sampling. This initial encounter is consolidated by formation of linker helix (LH)-D2 (residues 60–93) interactions with the CDK-cyclin module using an induced-fit mechanism (Fig. 2A).^34,35,63,68^

However, while the binding of p27 to A and D-cyclins is conserved, significant differences are found when comparing inhibitor binding to the kinase subunit in the CDK2-cyclin A-p27 and CDK4-cyclin D1-p27 structures (Fig. 2B).^37,69^ p27 binding results in burial of Tyr88 located in its 3_10_ helix (within the D2 region) into the CDK2 ATP binding pocket blocking ATP binding and inhibiting CDK2-cyclin A kinase activity. In comparison, the 3_10_ helix of p27 is not visible in the active site of CDK4-cyclin D1-p27 most likely due to steric hindrance by the hinge region of CDK4 (Fig. 2B).

Rotation within the CDK4 N-lobe in CDK4-cyclin D1-p27, relative to CDK4-cyclin D1, rearranges β-strands 2–5 to release the A-loop from the active site (Fig. 2B).^70^ Lys35 is repositioned from interacting closely with Asp158 to a position more compatible with ATP interaction. As these changes are not observed when p27 binds to CDK2, this appears to be a p27-mediated allosteric mechanism specific to CDK4. Overlay of the two complexes illustrates the difference in αC-helix positioning between the p27-bound and -unbound CDK4 structures. The N-lobe rotation of CDK4 now aligns with the N-lobe of CDK2 highlighting CDK4's slightly more favourable conformation for activation when bound to cyclin D1 and p27 (Fig. 2C). The rearrangement of the N-lobe, and in turn residues within the active site pocket, may provide a structural explanation for the desensitisation of CDK4 to current CDK4/6 clinical inhibitors palbociclib, ribocliclib and abemaciclib when CDK4 is present within the trimer complex.^69^

p27 phosphorylation also distinguishes CDK2 and CDK4. Site-specific phosphorylation triggers the dissociation of p27 from CDK2-cyclin A/E.^71,72^ p27 Tyr88 phosphorylation by Src-family tyrosine kinases is required for removal of the 3_10_ helix from the CDK active site. p27 binding is further weakened by phosphorylation of Tyr74 and Tyr89. These events decrease the ability of p27 to inhibit CDK2 and promote kinase activation and subsequent intra-complex p27 phosphorylation on Thr187.^65^

Phosphorylation of Tyr74 is a requirement for p27-mediated CDK4-cyclin D1 activation.^69^ However, in apparent contrast to CDK2,^65^ there was little additional kinase activity observed when the activity of the p27-triple mutant (Tyr74Glu/Tyr88Glu/Tyr89Glu)-bound CDK4-cyclin D1 was compared to that of the p27 single Tyr74Glu mutant-CDK4-cyclin D1 complex.^69^ The observed disorder in the CDK4-cyclin D1-p27 structure beyond Tyr74, would also support a model in which Tyr88 fails to adopt an ordered catalytic cleft binding mode as observed in the CDK2-bound structure (Fig. 2C). p21 Phe63 corresponds to p27 Tyr74, an amino acid change from which it has been hypothesized that p21 acts more as an assembly factor than as an allosteric activator for CDK4-cyclin D complexes.^73^ However, the αC-helix in CDK4 still remains out in both the CDK4-cyclin D1-p27 and CDK4-cyclin D1-p21 structures suggesting that the structure might be a snapshot compatible with crystallisation of intermediate-inactive states. Alternatively, the structure may be primed for substrate binding that, accompanied by further allosteric changes, promotes formation of the Michaelis complex. These experiments illustrate the distinct ways in which CDK4 and CDK1/2 respond to cognate cyclin association, and how subsequent phosphorylation and CIP/KIP association impacts activity. Further structural studies will be required to determine whether CDK6 resembles CDK4 or CDK1/2 in its response to binding of an authentic cyclin partner, and whether allosteric regulation by p21 and/or p27 is conserved with CDK4. Taken together structural studies to date suggest a CDK4-specific mechanism of activation, and by extension a range of structurally distinct activation states for all the cell cycle CDKs that might be distinguished by small molecule binding. It remains to be seen whether exploiting the differences by which their activities are regulated can be exploited to generate CDK-specific allosteric inhibitors.

Non-canonical CDK-cyclin complexes may extend this source of structural diversity to distinguish CDKs, and where they result from aberrant protein overexpression may offer cancer cell-specific opportunities for allosteric inhibitor design. As an example, three groups recently identified CRL4^AMBRA1/DCAF3^ as the E3 ubiquitin ligase that targets D-type cyclins for degradation *via* the ubiquitin proteasome pathway.^74–76^ Loss of CRL4^AMBRA1/DCAF3^ leads to elevated cyclin D expression and the formation of CDK2-cyclin D complexes.^75,76^ Further analysis will be required to determine how D-type cyclins activate CDK2 and whether the complex is structurally distinct.

The INK4 family of proteins are CDK4/6 inhibitors, of which mutation, deletion and amplification can drive tumorigenesis.^8,66,77–79^ CDK6 upregulation can trigger increased levels of CDK6-INK4 complexes, which present significant resistance to clinical inhibitor treatment.^78^ In this study, mutations at the CDK6-INK4 interface or INK4 knockdown restored CDK4/6 inhibitor sensitivity. Computational modelling suggested a resistance mechanism in which changes to the CDK4 ATP binding site induced by INK binding to CDK4-cyclin D generated an active site less pre-disposed to bulkier ATP-competitive inhibitor binding but retaining residual ATP binding. In a separate AML study, CDK4 sensitivity to both ATP and palbociclib binding was reduced in the presence of high p16^INK4a^ levels.^80^ Collectively, these studies suggest a further mechanism of ATP-competitive inhibitor resistance: Increased INK4 expression generates CDK4/6-cyclin D-INK4 complexes that retain sufficient kinase activity to drive cell cycle progression but escape orthosteric small molecule inhibition. Overall, there is an emerging body of evidence pointing to more common amplification, deletion and mutation in CDK regulators rather than cell cycle CDKs themselves in driving inhibitor resistance. These mechanistic insights can be utilised with recent structures to guide rationale design of novel PPI inhibitors.^69,80,81^

## 3. CDK-containing macromolecular complexes

The determination of structures of large CDK-containing macromolecular assemblies has progressed significantly with advances in cryo-electron microscopy (cryo-EM). These structures can identify allosteric mechanisms to regulate CDKs, and to target CDK roles beyond their kinase activity. They also allow assessment of the structural diversity of populations of CDK molecules that when observed in solution and unconstrained by crystal lattice contacts may identify novel CDK conformations for inhibitor development.

The cryo-imaging of CDK-containing complexes, HSP90-CDC37-CDK4,^82^ APC/C^Cdc20^-CDK2-cyclin A2-CKS2,^83^ TFIIH and yeast CKM^84^ have previously been reviewed.^9,85^ Given the structural changes that accompany CDK4 binding to HSP90/CDC37, how CDK4 and CDK6 engage with this system continues to be of interest for drug discovery. Briefly, within the HSP90-CDC37-CDK4 complex the N-terminus of CDK4 is partially unfolded and intertwines with HSP90, whereas the C-terminal lobe is stabilised by CDC37 mimicking native hydrophobic and hydrogen bonding interactions typically observed between the two kinase lobes.^82^ This complex sequesters CDK4 until it is relinquished to other protein partners when correct re-folding of CDK4 drives release. Held within the HSP90-CDC37 pathway, kinases are resistant to ATP-competitive inhibitor binding, and so the pathway offers a further mechanism by which some kinases (such as CDK4) can escape orthosteric inhibition. The interaction of CDK4 with HSP90/CDC37 has also recently be exploited *via* the design and generation of peptides that mimic short unfolded regions within monomeric CDK4 that interact with HSP90.^86^ Both CDK4 peptides were shown to compete with CDK4 to inhibit binding to HSP90 and induce apoptosis. Further investigation of this interaction by structural and biochemical approaches would help to elucidate the mechanisms that determine selectivity of chaperone-client PPI inhibition and in this instance how it regulates CDK activity in both normal and inhibitor-resistant environments.

The HSP90-CDC37 system has been reported to distinguish forms of CDK6 differentiated by their thermostability.^87^ CDK6 when strongly bound to HSP90-CDC37 is more thermally unstable and potently targeted by inhibitors/degraders. A more thermostable form which only weakly associates with HSP90/CDC37 is resistant to inhibitors. A structural understanding of the mechanisms involved might aid future CDK6 inhibitor development.

Short linear motifs (SLiMs) found in intrinsically disordered regions of proteins are critical to CDK substrate selection and the timely regulation of their activity.^9,88^ They also mediate CDK-cyclin module integration into larger protein assemblies. Recent CDK-containing structures determined by X-ray crystallography and cryo-EM are now elaborating their binding modes and highlighting the importance of the cyclin as a SLiM recognition module. The CBFs present docking sites for SLiMs located in substrates and regulators. In contrast, their unstructured termini encode SLiM motifs that regulate their activity by controlling their stability and cellular localisation.

**Fig. 3:**
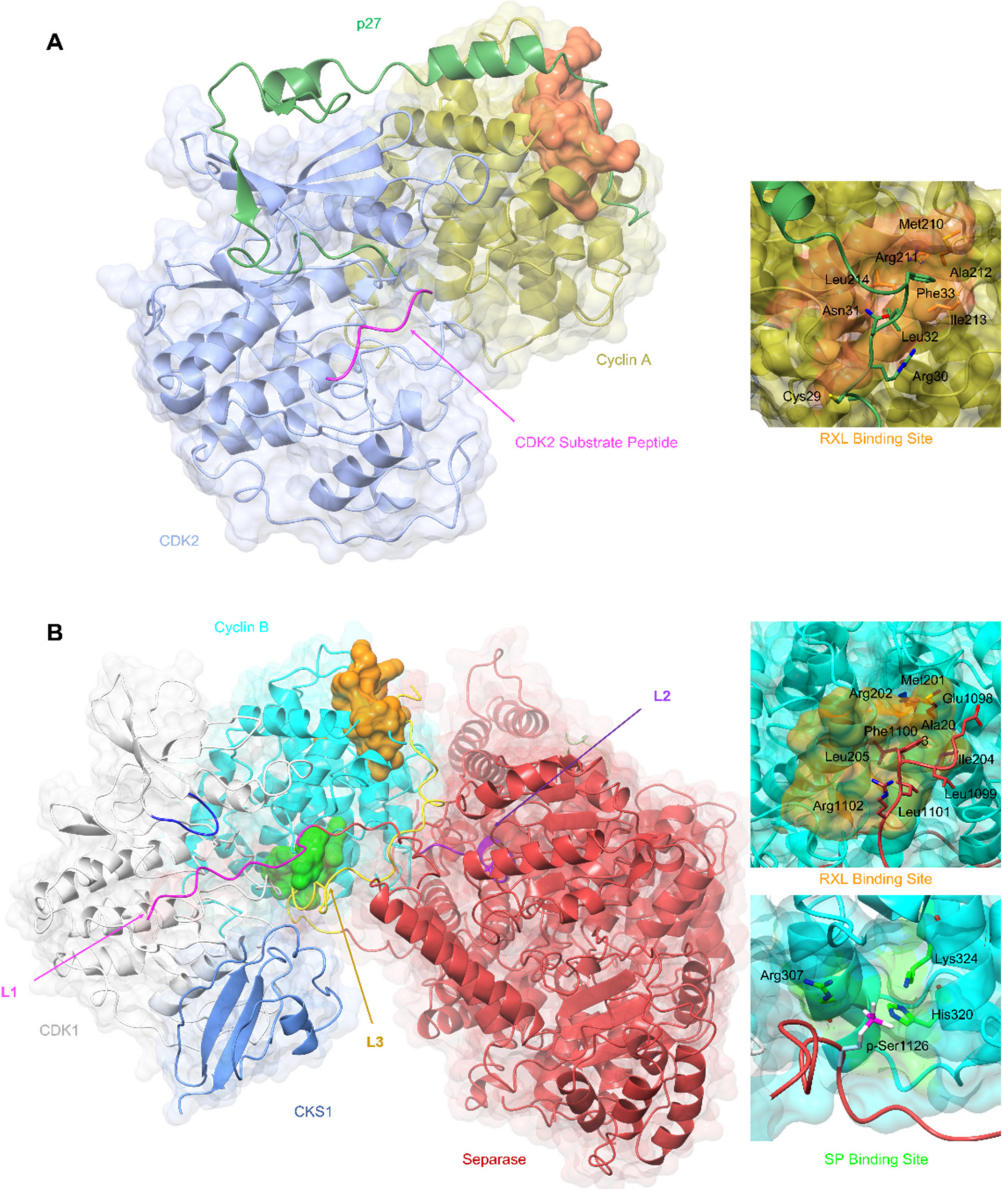
SLiM docking sites evidenced in the structures of CDK2-cyclin A-p27 and CDK1-cyclin B-Separase-CKS1. (A) Crystal structure of substrate binding to CDK2-cyclin A superposed with p27 from the CDK2-cyclin A-p27 structure. The CDK2 optimal peptide substrate peptide HHASPRK contains a conserved SP motif and is required for CDK2 substrate recognition and phosphorylation. (PDB: 1QMZ;^91^ CDK2-blue, cyclin A-gold, HHASPRK peptide – magenta). p27 illustrates a second SLiM interaction, the RXL motif - a short interacting motif present in substrates that bind to a pocket on the N-terminal lobe of cell cycle cyclins indicated by the MRAIL sequence. Binding is driven *via* the docking of an RXL motif into the hydrophobic patch on cyclin A (orange) as exemplified by the CKI, p27. (PDB: 1JSU;^67^ CDK2-blue, cyclin A-gold, p27-dark green). (B) Cryo-EM structure of CDK1-cyclin B-Separase-CKS1. (PDB: 7NJ0;^100^ CDK1-white, cyclin B-aqua, separase-red, CKS1-cornflower blue). Separase loop (L1, pink) binds adjacent to the CDK1 active site and blocks substrate docking and subsequent phosphoryl transfer through a shift in the G-loop (dark blue). This loop resembles that of the CDK2 substrate as illustrated in panel A. This loop acts as an inhibitory peptide, binding an SP-like motif but contains no residues compatible with CDK phosphorylation. Separase loop 3 (L3, yellow) docks SLiMs at two sites on cyclin B. L3 contains a typical RXL-like motif and binds the hydrophobic patch on cyclin B (orange). Separase L3 binding reveals a phosphate binding pocket (green) between H1′–H2′ on the cyclin B C-lobe which binds a second phosphorylated SP motif. Arg307, His320 and Lys324 form a positively charged region for electrostatic interaction with the separase p-Ser1126.

One of the first CDK SLiMs to be studied was the RXL motif found in a range of CDK substrates (for example E2Fs, p53, and pRB pocket proteins) and p21/p27 inhibitors.^65,66,89,90^ The cyclin RXL binding site was first exemplified by the structure of CDK2-cyclin A-p27 (PDB 1JSU^67^) and subsequently termed the cyclin recruitment site or hydrophobic patch (Fig. 3A).^67,91^ Recent work has identified several SLiMs that bind to the N-terminal cyclin box fold (N-CBF) within cell cycle cyclins that suggest that within this cyclin subset a significant percentage of this fold's surface is exploited in this way.^92–94^ Conserved sequence differences between cell cycle cyclins and between docking motifs combine to generate overlapping interactions of varying affinity. This structural variety provides opportunities for competitive protein binding and a mechanism to tune the timing of CDK substrate phosphorylation and integrate signalling pathways.^95^ Characterisation of the binding of SKP2 (S-phase kinase associated protein) to cyclin A has revealed how cyclin recruitment site promiscuity for binding SLiMs is increased by being able to recognise sequences in a “reverse” binding mode^96^ and how mutations can both dial up and down affinity for competing proteins, in this example SKP2 *vs.* p27.^97^

Cyclin B has provided the first example of a SLiM binding site on the C-terminal CBF (C-CBF) surface of a cell cycle cyclin. CDK1-cyclin B is the master mitotic regulator and phosphorylates many mitotic proteins to effect substantial cellular reorganisation to facilitate chromatid segregation and cytokinesis. In early mitosis, chromosomes are structurally restricted within a ring-shaped cohesin complex.^98^ Separase cleavage is required for chromosomal segregation upon degradation of its inhibitors, securin and cyclin B.^99^ The separase-CDK1-cyclin B-CKS1 (CCC) complex recognises and orders three inhibitory motifs each found in a flexible separase loop (Fig. 3B).^100^ The first autoinhibitory loop (L1) comprises the pseudosubstrate SLiM APxxxxR (Pro1375-Arg1386) which resembles that of a consensus (S/TPXXK/R) CDK phosphorylation site motif.^101,102^ This loop mimics the binding of a consensus CDK substrate into the CDK active site. Overall, the CDK1-cyclin B module largely conforms to an active kinase complex as evidenced by a DFG-in conformation. However, unlike a bone fide peptide substrate, the separase L1 loop also contacts and reorganises the CDK1 glycine-rich loop (G-loop) to sterically hinder the active site thus blocking CDK1-cyclin B activity.

An extended loop in the separase sequence (L3) spans both CBFs of cyclin B and makes specific SLiM-mediated docking interactions on each. The N-CBF interaction site is the RXL recruitment site that docks residues 1098–1103 encoding the ELFLRG RXL-like sequence (Fig. 3B).^100^ The cryo-EM maps show unambiguous density beginning at residue 1098. The second site within the C-CBF recognises a phosphorylated SP motif (Fig. 3B). This novel cyclin B phosphate binding pocket is forged from a triad of hydrogen bonding residues, Arg307, His320 and Lys324, located within helices H1 and H2 in the C-CBF. Phosphorylation of separase Ser1126 by CDK1-cyclin B is proposed to kickstart loop ordering by driving loop docking into this site.

Many CDK1 substrates contain multiple SP motifs and this structure could highlight one possible mechanism by which CDK1-cyclin B complexes achieve substrate specificity by offering an additional phosphorylated substrate binding site.^103^ Such a mechanism is precedented by CKS1 which also has a structurally characterised phospho-Ser/Thr binding pocket and when bound to CDK1 promotes multi-site substrate phosphorylation.^104,105^ The phosphate binding site is conserved in cyclin B but is not present in other cell cycle cyclins offering a possible explanation for the specificity of cyclin B for separase binding and its ability to coordinate its timely release in late metaphase.

Extended linear protein docking sites and phospho-Ser/Thr binding pockets are not the most tractable to target with small molecules. As discussed below the implementation of fragment screens against structurally enabled CDK regulators that exploit SLiMs may provide an opportunity to identify key interaction sites within extended sequences that drive potency and may be starting points for probe design. Peptidomimetics starting from cyclin-peptide complex structures also offer opportunities for inhibitor development.^106,107^

Cyclins A and B have extended unstructured N-terminal sequences that contain multiple regulatory sequences and phosphorylation sites. Two E3 ubiquitin ligase complexes, the anaphase-promoting complex/cyclosome (APC/C) and the Skp1-Cul1-F-box (SCF) protein complex, orchestrate the ubiquitination and subsequent degradation of many cell cycle regulators including these cyclins by recognition of SLiMs. Cyclin A2 and cyclin B1 are substrates of the APC/C pathway.^83,108^ APC/C selects substrates through two coactivator subunits, Cdc20 and Cdh1 which recognise conserved SLiMs including the destruction box (D box), KEN box and ABBA motif.^109–111^ Following cyclin A2 degradation, successful completion of chromosomal alignment signals SAC inactivation, and exchange of Cdc20 for Cdh1 that then promotes rapid ubiquitination of cyclin B1 by APC/C.^108^

Molecular interactions between APC/C^Cdc20^ and cyclin A2 have been characterised from the cryo-EM derived structure of the APC/C-cyclin A2 complex (PDB: 6Q6G).^83^ This structure captures two degron SLiMs, D1 and a novel degron box D2, which influence the formation of two distinct structural classes of cyclin A2-APC/C^Cdc20^ binding. The D1 degron bound to Cdc20 shows a poorly resolved elongated kinked loop density at the D-box binding domain. The D2 box bound to Cdc20 shows a canonical well resolved bridge between Cdc20 and APC10, stabilising binding to the APC/C. With D2 engagement, poor density for the KEN box and ABBA motif is observed. This stabilised binding renders the D2 box self-sufficient in the ubiquitination of cyclin A2 though its activity is enhanced by the ABBA and KEN motifs. In contrast, D1 box requires concerted binding of both the KEN and ABBA boxes.

Other cell cycle CDK binding proteins which are specifically recognised by E3 ubiquitin ligases and for which structural models exist include p27, and cyclins D and E recognised by the F box containing proteins SKP2 (in conjunction with CKS1^112^), FBXO31,^113^ and FBW7^114^ respectively. Each substrate contains a SLiM, and F box recognition is phosphorylation dependent in the cases of p27 and cyclin E. Of these three examples, there has been activity to identify small molecule inhibitors of the p27-SKP2/CKS1 interaction,^115,116^ where the p27 binding site is more tractable to small molecule binding.

### 3.1. Cryo-EM to elucidate structural mechanisms regulating the transcriptional CDKs

Members of the CDK family that regulate transcription are found in structurally diverse large assemblies that have historically proved challenging for structure determination.^117–122^ Recent advances in cryo-EM however have made these assemblies tractable to structural analysis and have revealed both alternative routes to CDK activation and additional mechanisms by which the cyclin coordinates the activities of CDK regulators. Targeting CDKs 7, 8 and 9 with ATP-competitive inhibitors has met with mixed success in the clinic. Given their fundamental roles in controlling gene expression, identifying settings in which substrate-specific CDK phosphorylation events drive tumorigenesis might offer opportunities for therapy.^49,123–127^ For example, some cancers have acquired sensitivity toward transcription inhibition, in particular those driven by super-enhancers and oncogenic transcription factors, such as RUNX1 and N-MYC.^33,128–132^

**Fig. 4:**
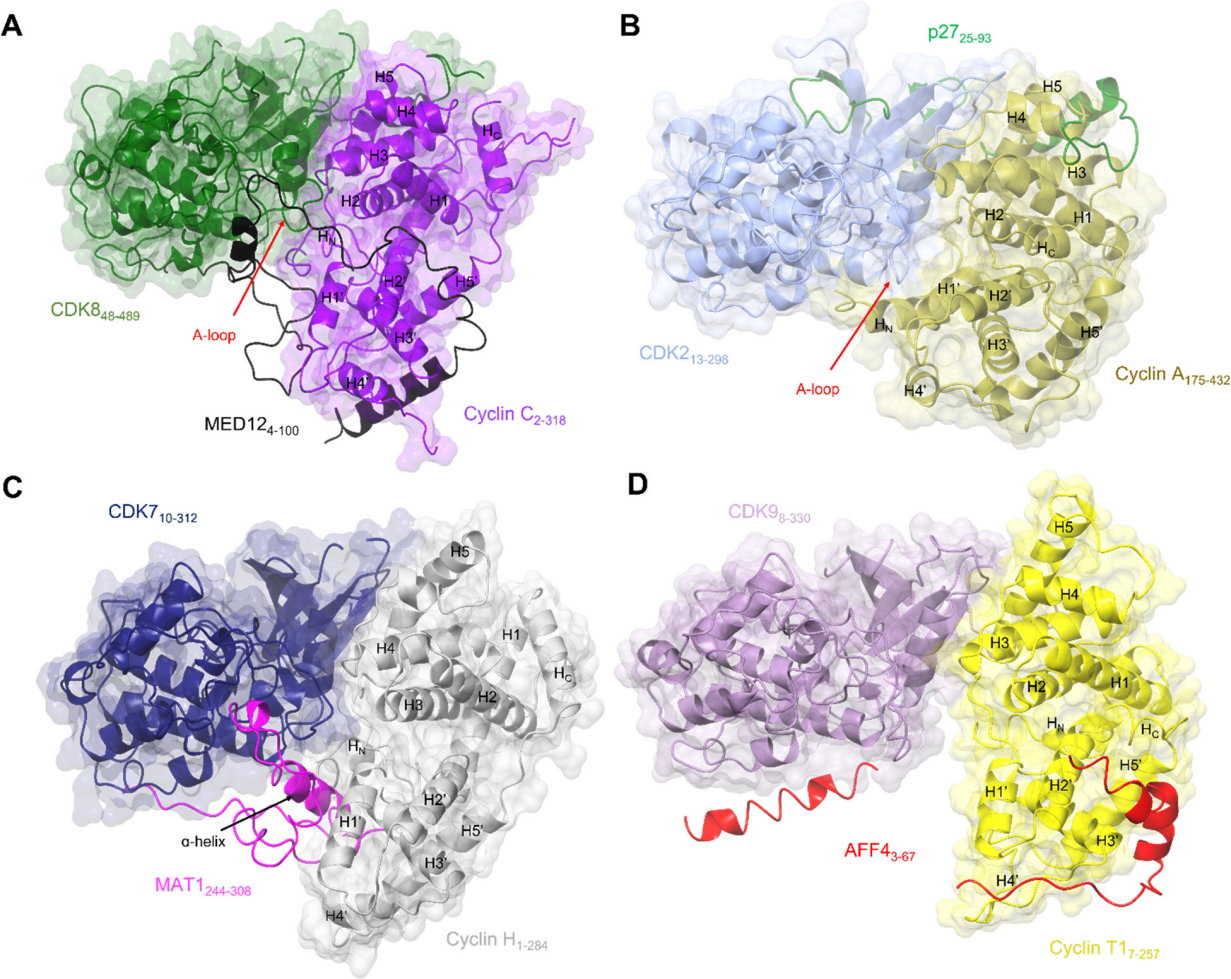
Comparison of the transcriptional CDK-cyclin complexes with the cell cycle CDK, CDK2-cyclin A. Comparison of the transcriptional CDK-cyclin complexes, for which a third binding partner has been identified and structures are available, highlights the differences in the CDK assembly and exploitation of the splayed cleft by an additional binding partner. The CDK2-cyclin A interface is tight in comparison to transcriptional CDK complexes thus partner binding is confined to the N-lobes. The splayed cleft of transcriptional CDKs offer additional binding surfaces for a partner protein between the C-lobes for a third partner protein between the C-lobes. (A) Cryo-EM structure of yeast CKM (PDB: 7KPX;^134^ CDK8-green, cyclin C-purple and MED12 - black. MED13 is omitted from this structure). (B) Crystal structure of CDK2-cyclin A-p27 (PDB: 1JSU;^67^ CDK2-blue, cyclin A-gold and p27-green. (C) Cryo-EM structure of CDK7-cyclin H-MAT1 (PDB: 6XBZ;^133^ CDK7-navy, cyclin H-grey, MAT1-pink). (D) Crystal structure of CDK9-cyclin T1-AFF4 (PDB: 4IMY;^137^ CDK9-lilac, cyclin T1-yellow, AFF4-red).

The structures of transcriptional CDK-cyclin complexes, CDK7-cyclin H (PDB: 6XBZ),^21,133^ CDK8-cyclin C (PDB: 7KPX),^134^ CDK9-cyclin T (PDB: 3BLH, 3MIA, 4IMY, 4OGR, 5L1Z, 6CYT)^135–140^ and CDK12-cyclin K (PDB: 4NST)^141^ and CDK13-cyclin K (PDB: 5EFQ)^142^ are characterised by a more splayed disposition of the CDK and cyclin subunits than is seen in for example CDK2-cyclin A (PDB: 1JST, 1JSU)^37,67^ (Fig. 4). For these CDK-cyclin modules, the complex is stabilised almost exclusively by interactions between the N-terminal lobes of each protein creating a cleft between the C-terminal lobes. Exploitation of this cleft by regulatory proteins was first exemplified by a series of complexes containing CDK9-cyclin T bound to AFF4, a scaffolding component of the super elongation complex.^137,138^ AFF4 is an intrinsically disordered protein, encoding multiple SLiMs. Though the N-terminal AFF4 sequence cannot be traced throughout its length, the electron density maps resolved sequences (residues 34–69) binding to the cyclin T C-CBF. In some structures, (though stabilised by crystal packing) AFF4 helix 0, (residues 4–21) can be resolved as binding to the CDK9 C-terminal lobe (Fig. 4D).^137,138^ Although the central AFF4 sequence of this N-terminal fragment is unresolved (residues 22–32), it likely bridges the gap between the CDK and cyclin subunits. Where Tat is present, the N-terminal region of its activation domain binds to the A-loop of CDK9.

Exploitation of this cleft between the CDK and cyclin subunits has now also been observed in CDK8 and CDK7, following the resolution of complex structures by cryo-EM. The CKM is composed of CDK8 (or its vertebrate-specific paralog CDK19), cyclin C, and mediator complex subunits MED12 and MED13.^134^ This module associates with the core mediator complex (cMED), an assembly of up to 26 proteins, to form the Mediator complex.^143,144^ The first crystal structure of CDK8-cyclin C bound to sorafenib (PDB 4F7S)^145^ revealed features that suggested a malleable kinase fold amenable to Mediator protein regulation. Notably the CDK8 A-loop does not contain a canonical phosphorylated residue and beyond the DMG motif it was predominantly disordered. Sequences below the A-loop (residues 239–247) that compose the αF-αG linker sequence were also disordered, suggesting a requirement for further protein binding in addition to cyclin C association to order this face of the kinase.

As revealed by a subsequent cryo-EM structure^134^ of the *S. cerevisiae* CKM complex, MED12 adopts a scaffolding function in which it binds to both cyclin C and CDK8 to bridge the CDK-cyclin module to MED13 that in turn associates the CKM and the cMED. The MED12 N-terminal helix and its flanking sequences (residues 35–56) are responsible for CDK8-cyclin C activation by inducing an active kinase conformation in which the DLG motif (DMG in humans) adopts an “in” conformation as seen in CDK2-cyclin A and the A-loop is stabilised (Fig. 4A).^134,146^ The importance of maintaining the integrity of the CDK8-MED12 interface for CDK8 activity is evidenced by the number of mutations at interfacial residues that disrupt MED12 binding and CDK8 activation^147^ and are associated with various diseases and errors in development.^134^ Mutations outside the interface also disrupt the ability of MED12 to activate CDK8. From this observation it has been proposed that these external mutations reconfigure the A-loop into a conformation that is not compatible with substrate phosphorylation, however functionally important scaffolding functions are retained. These studies reconfirm the conformational flexibility leading to kinase activation as seen in the crystal structures of CDK8. They also rationalise the effect of clinically significant mutations that may identify potential sites for inhibitor development.^134^

Structural features of CDK8 activation are conserved in CDK7, which also requires both a cyclin and a second partner (in this case the RING finger protein MAT1) for activation.^22,148,149^ Structures of this CAK module alone^21^ (Fig. 4C) and in complex with two ATP-competitive inhibitors, ICEC0942^133^ and THZ1,^21^ have been resolved by cryo-EM at high resolution. The CDK7 and cyclin H contacts are, as expected from the crystal structures, mostly confined to their N-terminal lobes, while their C-lobes are twisted away from each other to create a cavity that allows for near complete burial of the MAT1 C-terminal fragment (residues 255 to 309).^21,38,135^ These structures support the earlier biochemical observations that MAT1 increases CDK7-cyclin H stability through bridging of the two units. They also help rationalise early structures of TFIIH in which only the N-terminal fragment of MAT1 was resolved.^25,84,150^ These structures revealed TFIIH subunit assembly and the bridging of the XPB and XPD ATPase/helicase subunits by MAT1. However, they were unable to fully resolve the CDK7-cyclin H module and connecting MAT1 residues (210–244) due to disordered regions and/or ambiguous density.

The most prominent feature is the MAT1 C-terminal α-helix (residues 288–300) which spans the breadth of the cleft to contact both CDK7 and cyclin H. The additional helix runs antiparallel to the H1’-helix in cyclin H and occupies a similar position to that of the extended H_N_ α-helix present in the cyclin subunits of CDK2-cyclin A, B and E and CDK1-cyclin B complexes (PDBs: 1FIN, 4YC3, 2JGZ, 1W98, 6XBZ, respectively).^21,38,59,63,151^ This extended helix is not seen in the structures of CDK8-cyclin C and CDK9-cyclin T1.^40,70,145^ However, in cryo-EM structures of the yeast CKM (Fig. 4A), the N-terminus (residues 4–58) of MED12 occupies the space between CDK8-cyclin C.^134^ A short helix (residues 41–48) contacts the C-lobe of CDK8 and the preceding looped region (residues 32–38) runs identical to this extended helix alongside the activation loop.^134^

Together, these transcriptional CDK-containing complexes illustrate the variety of CDK-cyclin structures and strengthen the model that they have a conserved mode of regulation that exploits the cleft between them to bring regulators recognised by the cyclin into the vicinity of the kinase active site. As has been observed for the cell cycle CDKs, the use of overlapping and adjacent protein binding sites provides opportunities for CDK regulation and signalling pathway integration. As the size limit for cryo-EM structure determination drops, and as exemplified by structures of CDK7 bound to the ATP-competitive inhibitors ICEC0942^133^ and THZ1,^21^ it is now possible to consider this method as part of a drug discovery pipeline. Though throughput for the moment will not match X-ray crystallography, the ability to capture native conformationally distinct protein populations and analyse allosteric binding modes offers exciting new opportunities for both orthosteric and allosteric CDK inhibitor design.

## 4. Advances in structure determination to develop CDK inhibitors that exploit protein degradation pathways

Recently, the ubiquitin proteasome system (UPS) has been utilised to generate novel therapeutics that induce the degradation of CDKs and cyclins. Of particular interest is the potential of this approach to target proteins previously considered “undruggable” because they lack suitable or selective binding sites for small molecules, or they present experimental challenges to traditional compound screening methodologies. Compounds that fall into this category include non-peptide heterobifunctional molecules, referred to as proteolysis targeting chimeras (PROTACs), and molecular glues.

PROTACs contain two protein-binding moieties connected by a linker. One ligand binds to the protein target and the other binds to an E3 ligase of which Cereblon (CRBN), von-Hippel lindau (VHL) and inhibitors of apoptosis proteins (IAP) complexes are commonly used. Their advantages include ubiquitous expression and having well-characterised interacting ligands. The ternary complex formed upon binding to both interaction partners hijacks the UPS to degrade the target. The effectiveness of this process is reliant on, but not limited to, the unique ternary complex generated by the individual target-specific ligands, the choice of E3 ligase and the linker composition and orientation.

**Fig. 5:**
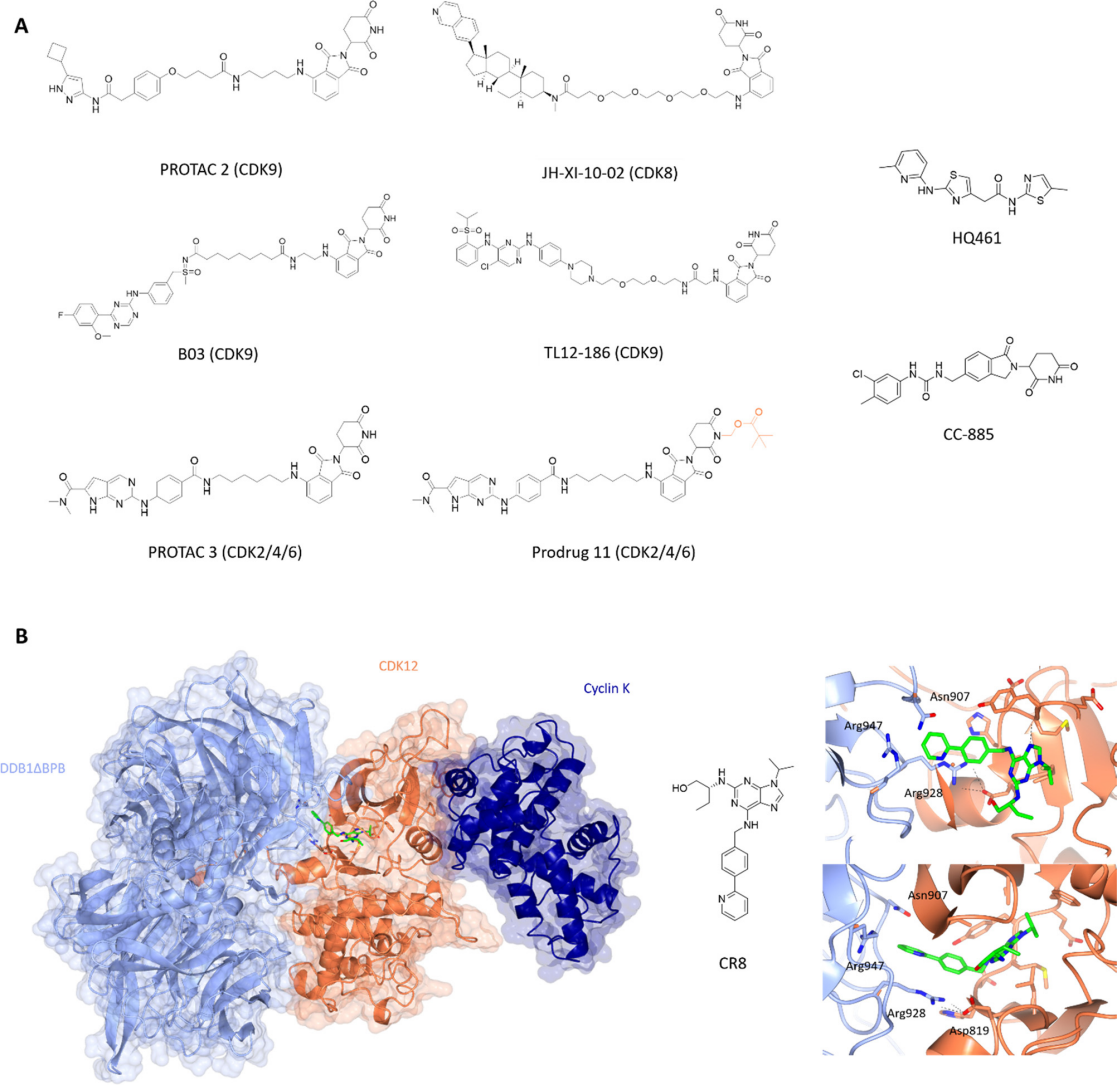
Design and structural validation of CDK PROTACs and molecular glues. (A) Chemical structures of example CDK PROTACs designed from both pan and specific CDK inhibitors. PROTAC 3 was developed into the bio-orally available Pro-drug 11 *via* the addition of a pivalate substituent shown in orange. HQ461 and CC-885 are examples of current CDK molecular glues.^153,156,164,170,171,175,179^ (B) CDK12-cyclin K complexed with DDB1 *via* the molecular glue CR8 (PDB: 6TD3;^178^ CDK12-orange, cyclin K-dark blue, DDB1-pale blue). The CDK12 active site is occupied by CR8 which extends the hydrophobic phenylpyridine towards DDB1 to interact with residues Asn907 and Arg947 of the nearby DDB1 interface. The C-terminal tail of CDK12 extends into the cleft between the DDB1 lobes further stabilising the interaction. Cyclin K is bound to the opposite side of CDK12 in the classical CDK-cyclin conformation.

CDK9 remains an attractive target for therapeutic intervention, but the failure of many CDK9 small molecule inhibitors to progress through to the clinic has been a driver to explore PROTACs as an alternative approach. The first published CDK9-targeting PROTAC consists of an aminopyrazole-derived ligand chemically linked to thalidomide, a cereblon (CRBN) ligand.^152^ This pan-CDK inhibitor core scaffold mimics ATP and makes hydrogen bonds between the aminopyrazole scaffold and the hinge backbone. The phenyl ring being solvent exposed provides a suitable handle for linker addition with negligible loss in activity. Various SAR substitutions resulted in degrader 3 with selectivity towards CDK9 at low μM efficacy with no significant effect on CDK2 or CDK5 levels. Optimisation of this PROTAC generated a CDK9-selective PROTAC with sub-μM efficacy with a DC_50_ of 158 ± 6 nM (Fig. 5A).^153^ Degradation of other CDKs was not observed despite similar IC_50_ values suggesting that the capacity of a PROTAC to degrade the target does not track with the binding affinity of the target ligand moiety and therefore tracking SAR by measuring kinase inhibition *in vitro* may be misleading in early development.

Other CDK9-targeting PROTACs include THAL-SNS-032,^154^ wogonin-based PROTAC 11c,^155^ B03^156^ and dual CDK2/9 degrader F3.^157^ Selective CDK9 degradation *via* sub-stoichiometric concentrations by degrader THAL-SNS-032, designed from the pan-kinase CDK2/7/9 inhibitor SNS-032, suggests a potential advantage over traditional small molecules in overcoming toxicity induced by the need for high doses of small molecule inhibitors.^154^ Alternatively, the use of less potent but selective CDK9 inhibitors such as wogonin in degrader development also resulted in sub-micromolar degradation.^155^ Similarly, B03 with the CDK9 selective warhead BAY-1143572 showed CDK9-specific degradation with a DC_50_ of 7.6 nM (Fig. 5A). These findings highlight that potent degraders can be generated from lower affinity and/or less selective starting ligands and still induce nM degradation profiles and significant selectivity.

Further degraders designed using structure-rationalisation were generated based on the modelling of AT-7519^158,159^ (CDK1/2/4/6/9 inhibitor) and FN-1501^160^ (CDK2/4/6/FLT3 inhibitor) with the CDK2 structure 2VTH.^157,159^ Solvent exposed saturated heterocycles (piperidine in AT-7519; piperazine in FN-1501) provided ideal handles for linker attachment. Interestingly, degraders A9 and F9 which only differ in the choice of CDK-binding ligand contradict the selectivity profiles of the parent kinase-inhibitors providing evidence for the role of the linker and E3 ligase in manipulating the resulting PROTAC selectivity.

In addition to its role in transcription, changes in CDK8 expression have been linked to oncogenic survival associated with pathways that include Wnt- and TGF-β signalling.^161–163^ The first CDK8 PROTAC JH-XI-10-02 (Fig. 5A),^164^ was developed using analogue JH-VIII-49 connected to pomalidamide with a PEG_4_ linker. Treatment for 24 h at 1 μM resulted in substantial degradation of CDK8. This compound provides a good tool for investigating the effects of CDK8 chemical knockdown on an acute timescale where further characterisation and optimisation may lead to new CDK8-specific PROTACs as novel therapeutics.

PROTACs active towards cell cycle CDKs have also been identified. Teng *et al* reported the development of PROTACs from pan-inhibitor TMX-2039 with PEG/alkyl linkers connected to CRBN imide ligands.^165^ TMX-2172 induced selective degradation of both CDK2 and CDK5 at 250 nM. However, a reduction in Aurora kinase A and weak activity against 3 other kinases was also detected in wider-proteomic profiling.

CDK4 and CDK6 PROTACs all involve the use of clinical inhibitors palbociclib, ribociclib and abemaciclib as substrate binding ligands. PROTACs Pal-pom and Ribo-pom exerted better dose-dependent degradation of CDK4 (DC_50_ of 15 and 100 nM, respectively), but still displayed some degradation capabilities against CDK6,^166^ with a downstream reduction in pRB levels. Other CDK4/6 targeting degraders include imide-based PROTACs BSJ-02-162 and BSJ-03-204, consisting of Palbociclib linked to thalidomide by an alkyl chain, and only differ by an aryl amide nitrogen *vs.* oxylamide from thalidomide.^167^ At 1 μM, both PROTACs effectively degraded CDK4 and CDK6 with effects observed as low as 100 nM. Degraders such as BSJ-04-137 selective towards CDK4 and BSJ-03-123 with CDK6 selectivity provide good tools for differentiating between these CDKs. Further analysis confirmed selectivity to CDK4/6 as previous investigations had shown imide-based degraders also targeted IKZF1/3.^168^

BSJ-03-123 highlighted that global transcriptional and signalling effects of CDK6 are mainly *via* its kinase activity.^169^ In AML cell lines BSJ-03-123 efficacy was dependent on INK4 status.^81^ As previously mentioned, p16^INK4a^ has been shown to confer resistance to palbociclib treatment when highly expressed, however p16 does not necessarily sensitise cells to palbociclib when at low levels. It was hypothesised that this INK4-dependent mechanism may also affect the ability of BSJ-03-123 to degrade CDK6. In the presence of high p16^INK4a^/p18^INK4c^ levels, CDK6 appeared to be protected from degradation by BSJ-03-123 but was still able to bind ATP. These observations suggest that under cellular conditions of high p16^INK4a^/p18^INK4c^, degraders are not as effective and may be more effective in combination with other treatments.

The latest advance in CDK2/4/6 PROTACs has been the development of a prodrug PROTAC with bioavailability.^170^ PROTAC 3 consists of ribociclib and pomalidamide with an amide linker (Fig. 5A), and produced significant degradation of CDK2, 4 and 6 in a dose-dependent manner below 1 μM. Cell cycle arrest at G0/G1 and G2/M consistent with loss of CDK2/4/6 activity was observed. However, despite this report, oral bioavailability remains a challenge for CRBN-PROTAC development.

An alternative application of PROTACs was used by Riching and colleagues where a pan-kinase PROTAC facilitated CDK family profiling in terms of kinetic degradation rates, Dmax and potency differences.^171^ The majority of CDKs analysed showed rapid and almost complete degradation after 24 hours. In agreement with earlier studies, 9 family members (mostly transcriptional or understudied CDKs) were identified as the most susceptible TL12-186 substrates in which the greatest extent of CDK degradation accompanied TL12-186 treatment.^172^ Use of a CRISPR-Cas9 HiBiT tagging system to permit live-cell kinetic analysis revealed a cell cycle phase specific CDK2 degradation profile.^171^ While CDK2 could bind TL12-186 in all cell cycle phases, ternary complex formation with CRBN was only observed during G1. This result suggests that interaction with CRBN and subsequent degradation may require a specific cellular location or structural state of CDK2 which is only present in G1-phase. Investigations such as this provide information about the temporal association and degradation of specific CDK complexes and reveals selective windows for successful inhibition/degradation of certain CDKs. They also highlight that structure determination of CDK-PROTAC-E3 ligase complexes is required to understand the apparent differences in activity.

### 4.1. Molecular glues and transcriptional CDKs

Molecular glues are small molecules which induce the degradation of neo-substrates by changing the surface of the substrate to promote E3 ligase interactions, resulting in ubiquitination and proteasomal degradation. This modality is unique to these molecules and only a few have been identified so far against CDKs. One of the unique properties of molecular glues is the ability to recognise and bind relatively flat or disordered protein surfaces, making these of interest to drug discovery programs against targets that lack identifiable pockets. After the discovery of auxin, a plant hormone which induces transcription factor degradation *via* E3 ligase CRL1TIR1,^173^ further molecules such as thalidomide and analogues lenalidomide and pomalidomide were developed against CRBN^174^ suggesting that this mechanism of action is not confined to plant systems and can potentially be manipulated for disease-state targets where current small molecules have failed.

Current molecular glues against CDKs are limited to CDK12, CDK13, and CDK4. HQ461 mediates the degradation of cyclin K by interacting with CDK12-cyclin K and the DDB1-CUL4-RBX1 E3 ligase complex.^175^ It has an aminopyridinylthiazole scaffold and relies on the 5-methylthiazol-2-amine for activity as reflected in the significant drop off in Dmax (10 μM at 8.6% and 9.9%) for analogues HQ014 and HQ015 respectively compared to 86% for HQ461. The addition of bulky ring substituents at the 5-position of the pyridyl also increased the degradation potency. HQ461 acts *via* direct interaction with CDK12 and is suggested to occupy the ATP-pocket as dinaciclib treatment blocks cyclin K degradation by HQ461. However, the structure of a HQ461-CDK12-DDB1-CUL4-RBX1 complex to confirm this mechanism of action is yet to be solved. The unique aspect of this complex is that HQ461 directly induces the interaction of DDB1 with CDK12, unlike other molecular glues which use a substrate-specific receptor protein known as a DCAF (DDB1 and CUL4 associated factor).^176,177^ Furthermore, CDK12 itself is not ubiquitinated highlighting a scaffold-like role in facilitating the degradation of cyclin K.

Similarly, work by Slabicki and colleagues identified CR8 as a molecular glue of CDK12-cyclin K.^178^ Cyclin K was the only protein identified to show consistent reduction upon treatment with CR8, which was blocked by treatment with proteasome inhibitor MG132, E1-activating enzyme inhibitor MLN7243 and neddylation inhibitor MLN4924. These results support CR8-mediated degradation of cyclin K as the mechanism of action for the cytotoxicity observed for CR8. In a similar manner to HQ461, CR8 binds to CDK12 to facilitate complex formation with DDB1-CUL4-RBX1 with nM affinity. Structural elucidation of CDK12_(713-1052)_-cyclin K_(1-267)_-CR8-DDB1ΔBPB (PDB: 6TD3)^178^ to 3.5 Å confirmed that CR8 occupies the CDK12 active site with its phenylpyridine moiety extending towards DDB1 (Fig. 5B). The CDK12 C-terminal tail interacts with the cleft between the DDB1 BPA and BPC domains.

The only CDK4 targeting molecular glue reported is CC-885.^179^ CC-885 is a derivative of lenolidamide and was initially identified as a degrader of GSPT1,^180^ Bnip3L^181^ and PLK1.^182^ Further screening of potential neo-substrates of this ligand showed CDK4 degradation in a CRBN-dependent manner accompanied by reduction in pRb and mRNA levels of E2F downstream genes.^179^ A further decrease in CDK4 levels was observed when combined with genetic or chemical (palbociclib) perturbation of CDK4. While CC-885 may not be the most potent ligand against CDK4, it is the first molecular glue identified against a cell cycle CDK and shows promise for further exploration with respect to selectivity and potency towards CDKs.

## 5. Fragment library screening against CDKs

The identification of ligand binding sites is critical to an assessment of protein druggability. Protein active sites have evolved to bind small molecules as co-factors and substrates making them primary targets for drug discovery campaigns. In contrast, allosteric sites are more difficult to identify and are often sites of protein–protein interactions.^183–185^ Fragment-based screening methods exploit simpler and smaller molecular scaffolds which can penetrate protein pockets.^186,187^ Improvements in potency to generate chemical probes and ultimately drug-like compounds can be achieved through fragment linking, merging or growing. This technique can also complement the rebuilding of current inhibitor scaffolds by identifying new chemical moieties with more favourable interactions.

**Fig. 6:**
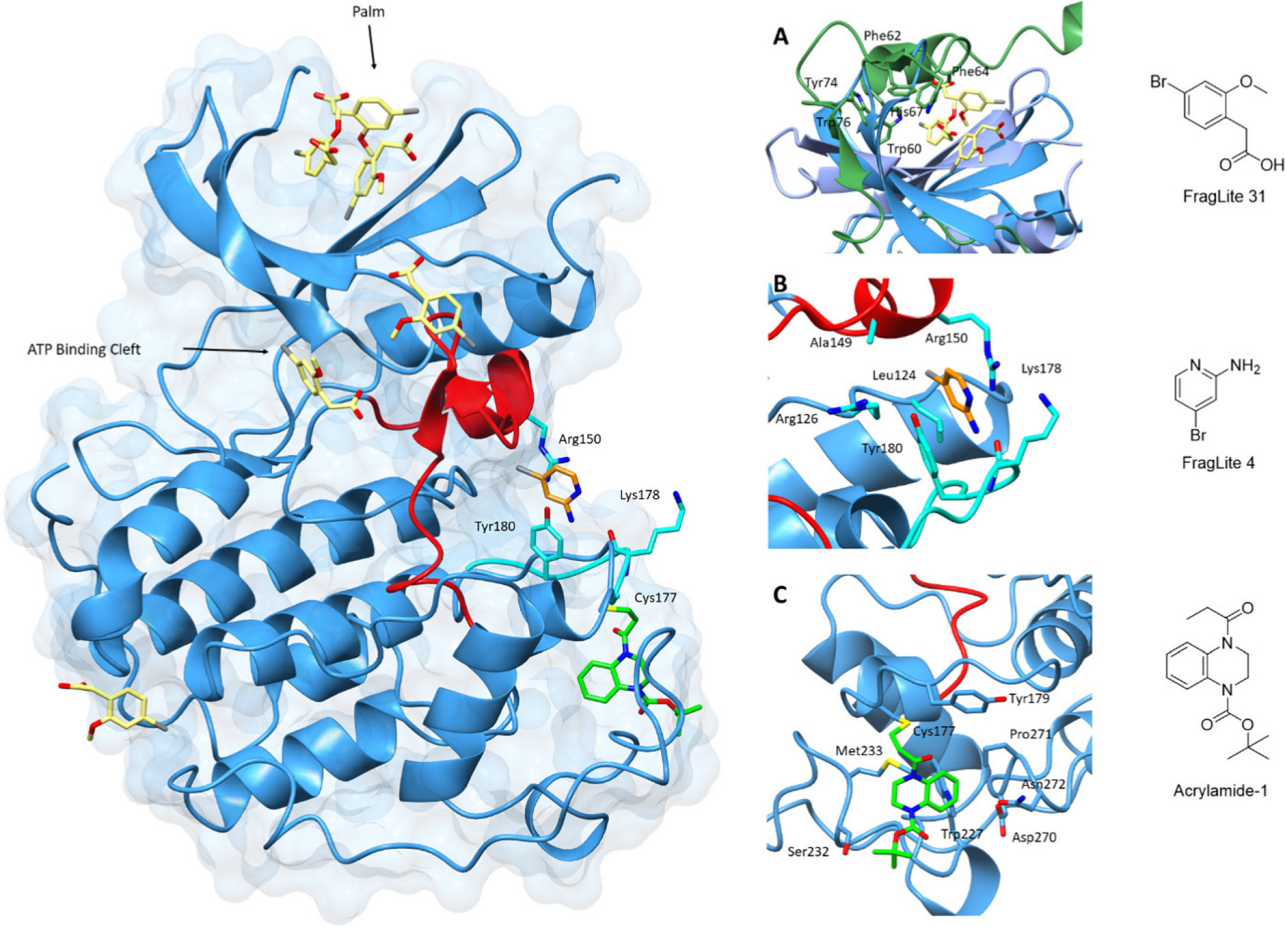
Fragment map of cyclin-dependent kinase 2. A map of fragment binding events on CDK2 reveals binding to the orthosteric ATP binding pocket and several pockets that overlap with the binding sites of known protein partners. Structural differences in the activation loop and connecting residues are shown for Acrylamide-1 (blue) and FL4 (cyan). (A) FragLite 31 (yellow) identifies the ATP binding cleft and three allosteric sites. Three copies of FL31 are buried in the palm region which mimics that of p27 which buries six aromatic residues in this region (PDB: 6Q4D).^184^ (B) FL4 (orange) occupies a site under the A-loop, overlapping the cyclin binding interface (PDB: 6Q3F).^184^ FL4 captures three types of intermolecular interactions with neighbouring residues Arg150, Lys178 and Tyr180 (cyan). (C) Acrylamide-1 (green) covalently binds to Cys177 at the cyclin binding interface (PDB: 5OSJ).^194^

Many fragment libraries are commercially available, exploring under-represented 3D space,^188^ pharmacophore cores,^189^ peptidomimetics^190^ and covalent electrophilic warheads.^191^ A small library of halogenated fragments known as FragLites has been shown to effectively map the interactome of CDK2 with relatively few fragments compared to traditional fragment libraries.^184^ The success of this library results from the improved visualisation of low occupancy ligand binding events provided by the halogen substituent. The greatest density of FragLite binding to CDK2 was within the ATP binding site, and through fragment elaboration a Type I inhibitor of μM potency was achieved. The successes of this library are also reflected in the ability to identify protein–protein interaction sites of regulatory protein partners and allosteric binding pockets. FL31 (PDB: 6Q4D)^184^ identified three such binding pockets on the surface of CDK2 with one of these sites binding three fragment copies (Fig. 6). An overlay with CDK2 structures reveals that this FL31 binding site in the N-lobe palm region is critical to the interaction of CDK2 with p27. However, FragLite binding to monomeric CDK2 cannot be directly compared with the crystal structure of CDK2-cyclin A-p27. p27 induces substantial reorganisation of the N-terminal CDK2 β-sheets to bury six aromatic residues in the CDK2 palm region alone. Multiple bound copies of FL31 reflect this preference for binding aromatic scaffolds.

A second interaction site as identified by FL4 (PDB: 6Q3F), superposes with a second PPI site, the cyclin binding interface.^184^ FL4 occupies a site below the A-loop which in this form adopts a DFG-out conformation. FragLite binding is supported through a π–cation interaction with Arg150, a π–π stacking interaction with Tyr180 and hydrogen bond donation to the backbone carbonyl oxygen of Lys178. This pocket provides experimental evidence for small molecule binding in an allosteric site which when occupied by pentameric peptides is proposed to break the CDK-cyclin interaction.^192^ A small fragment screen by Ludlow and colleagues identified an allosteric site which may also be used for allosteric inhibitor design that breaks the CDK-cyclin interaction further supporting the use of fragment libraries at identifying allosteric binding sites.^193^

This strategy of occluding the cyclin binding interface has also been explored through the use of covalent fragments. Cys177, a residue unique to CDK2, follows the A-loop and is positioned adjacent to the cyclin binding interface. Fragment-based screening of an electrophilic fragment library has identified the first CDK allosteric covalent inhibitor using quantitative irreversible tethering (qIT).^194^ Acrylamide 1 covalently modifies CDK2 (Cys177), exhibiting around 83% kinase inhibition, while the point mutant CDK2 (Cys177Ala) was unaffected. A crystal structure of CDK2-Acrylamide 1 confirmed covalent linkage to Cys177.

Fragment screening thus offers a route to identify potential sites of protein interaction on CDK-cyclin complexes. A comparative analysis between members of the CDK and cyclin families may highlight conserved sites that distinguish them. These sites may be amenable to downstream exploitation to identify protein–protein interaction inhibitors or warheads for the development of degraders for exploitation of the UPS.

## 6. Conclusions

With an increasing number of protein–protein and allosteric sites being identified through the structural exploration of larger CDK-containing macromolecular complexes, further understanding of protein–protein interfaces and their mechanisms of action can provide new sites to target. Recent studies have revealed the increasingly complex relationship between CDKs and their binding partners in regulating their activity and mechanisms of therapeutic resistance. Identifying sites necessary for key interactions with protein interaction partners and determining small-molecule binding capabilities *via* fragment screening may increase selectivity and reduce off-target effects typically observed for orthosteric inhibitors. ATP-competitive inhibitors have shown utility in PROTAC and molecular glue design. Whether targeting other pockets to identify more selective compounds through this approach has wider applications than the few examples reported to date is an exciting future prospect.

## Author contributions

I. H., J. A. E. and J. E. W. wrote the article, and I. H. and J. E. W. prepared the figures.

## Conflicts of interest

The authors declare that there are no competing interests associated with the manuscript. Some work in the authors’ laboratory is funded by Astex Pharmaceuticals.

## Supplementary Material
